# Inhaled Nitric Oxide Counterbalances ET-1 Dependent Pulmonary Hypertension and Bronchoconstriction in the Pig

**DOI:** 10.1155/S0962935194000165

**Published:** 1994

**Authors:** M. G. Clement, M. Dimori

**Affiliations:** Institute of Veterinary Physiology and Biochemistry University of Milan Via Celoria 10 Milan 20133 Italy

## Abstract

In anaesthetized, paralysed, ventilated pigs the ability of inhaled nitric oxide (80 ppm in 0_2_) to reduce the haemodynamic and respiratory effects of endothelin-1 administration (200 pmol/kg, i.v.) was evaluated. The mechanical properties of the respiratory system were evaluated by the rapid airway occlusion technique. The overall respiratory resistance, the interrupter resistance and the additional resistance that reflects the viscoelastic properties of tissues and the inequality of the time constant within the system were also evaluated. The results show that inhaled nitric oxide can act as a selective pulmonary vasodilator and as a bronchodilator to counteract the vasoconstrictor and bronchoconstrictor activity of endothelin-1. In the pig, nitric oxide inhaled at 80 ppm for 6 mitt reduced the changes in respiratory-, interrupter- and additional resistance due to endothelin-1 administration without significantly changing the static and dynamic elastance of the respiratory system.


Mediators of Inflammation 3, 131–135 (1994)

### Inhaled nitric oxide counterbalances ET-1 dependent pulmonary hypertension and bronchoconstriction in the pig

**M. G. Clement[CA] and M. Dimori**

Institute of Veterinary Physiology and Biochemistry, University of Milan, Via Celoria 10, 20133 Milan, Italy

[CA] Corresponding Author

**In anaesthetized, paralysed, ventilated pigs the ability of inhaled nitric oxide (80 ppm in $O_2$) to reduce the haemodynamic and respiratory effects of endothelin-1 administration (200 pmol/kg, i.v.) was evaluated. The mechanical properties of the respiratory system were evaluated by the rapid airway occlusion technique. The overall respiratory resistance, the interrupter resistance and the additional resistance that reflects the viscoelastic properties of tissues and the inequality of the time constant within the system were also evaluated. The results show that inhaled nitric oxide can act as a selective pulmonary vasodilator and as a bronchodilator to counteract the vasoconstrictor and bronchoconstrictor activity of endothelin-1. In the pig, nitric oxide inhaled at 80 ppm for 6 min reduced the changes in respiratory-, interrupter- and additional resistance due to endothelin-1 administration without significantly changing the static and dynamic elastance of the respiratory system.**

**Key words**: Bronchoconstrictor activity, Endothelin-1, Inhaled nitric oxide, Vasoconstrictor activity

### Introduction

There have been reports concerning mediators released from endothelium which modulate the contraction of the vascular and non-vascular smooth muscles. These mediators include some arachidonic acid metabolites ($PgI_2$, $TXA_2$), the vasoconstrictor peptides (endothelin) and endothelium-dependent releasing factor (EDRF), recently identified as nitric oxide (NO).[1–7] Experiments performed *in vivo* in the guinea-pig or *in vitro* on isolated guinea-pig and rabbit airway segments showed that endothelin-1 (ET-1), a peptide of endothelin family, induces bronchoconstriction.[8–10] The localization of ET-1-like immunoreactivity in the airway epithelium of rats and mice, associated with the activity of the peptide on the tracheobronchial tree indicate that ET-1 has a role in modulation of bronchial tone.[11,12] Nitric oxide, the new potent endothelial vasodilator factor, recently discovered by Furchgott and Zadawski,[3] inhibits the contraction *in vitro* of tracheal smooth muscles from man and the guinea-pig,[13–15] but has no effect on canine bronchial smooth muscle.[16] Even though its effects on bronchial smooth muscles differ for different muscles, nitric oxide is considered to be a mediator of a nonadrenergic noncholinergic (NANC) system regulating bronchial tone.[13,15] It has been shown in animal and human studies that inhalation of NO attenuates the hypoxic pulmonary vasoconstriction response and tromboxane mediated pulmonary vascular contraction.[17–19]

Therefore, an imbalance of the ratio of ET-1 to NO could contribute to the appearance of bronchospastic and pulmonary hypertensive disorders. The authors wished to see whether or not inhaled NO, diffusing throughout the airway epithelium, might act directly on underlying smooth muscles to counteract the ET-1 bronchoconstrictor response.

In this study the ability of inhaled NO to reduce the effects of ET-1 on the pulmonary and systemic vascular bed and on the elastance and resistances of respiratory system in anaesthetized, paralysed, mechanically ventilated pigs, has been evaluated.

### Materials and Methods

Six Large White pigs, of either sex, weighing 20 ± 2 (S.E.) kg, were used. They were sedated with 1% propionylpromazine hydrochloride (0.05 ml/kg i.m.) and anaesthetized with 15 mg/kg thiopental-sodium injected into the auricular vein, followed by infusion, drop by drop of 9 mg/kg/h. The animals were tied in the supine position on a heated operating table, tracheostomized, paralysed with pancuronium bromide (0.2 mg/kg, i.v.) and ventilated mechanically (Servoventilator Siemens 900 C). When necessary, additional paralysing drug (pancuronium bromide, 0.2 mg/kg, i.v.) was administered during the experiments.

Airflow ($\mathring{V}$) was measured by a pneumotachograph (Fleisch N.2) connected to a differential pressure transducer (Statham PM15,10846) and tidal volume ($V_t$) was obtained by electronic integration of $\mathring{V}$. Tracheal pressure ($P_{tr}$) was measured at the side port

© 1994 Rapid Communications of Oxford Ltd of the tracheal cannula by a differential pressure transducer (Statham PM15,15299). A Swan Ganz catheter (5F) was inserted into the pulmonary artery to monitor mean pulmonary arterial pressure (MPAP). The right femoral artery was cannulated with a polyethylene catheter to monitor mean arterial blood pressure (MAP). Systemic and pulmonary arterial pressure were recorded by connecting the catheters to a fluid-filled capacitance manometer (Bell & Howell 4-422). Haemoglobin, methaemoglobin and blood gas analyses were performed on an arterial blood sample (I.L.282 CO-Oximeter and Blood Gas Analyzer I.L.282). All parameters were recorded on a pen recorder (Nec San-ei Instruments Polygraph mod 8K40). The heart rate was evaluated by polygraph trace. $\mathring{V}$ and $P_{tr}$ were also recorded on an FM magnetic tape recorder (Racal Store) and, after sampling at 2 000 Hz by a 12-bit analogue-to-digital converter, analysed on an AST personal computer.

After evaluation of control values obtained when the animals were breathing air through the Servoventilator, ET-1 was administered through the Swan Ganz catheter at a dose of 200 pmol/kg. At the peak of ET-1 dependent vascular and respiratory effects (about 10 min), nitric oxide (NO) was inhaled. NO was administered through Servoventilator for 6 min. The inspired gas was a precise mixture of oxygen and nitrogen immediately diluted with NO to produce the desired concentration of inspired NO (80 ppm). With volumetrically calibrated flowmeters, NO in the bag (mixture of 235 ppm NO in pure $N_2$) was substituted for pure $N_2$ to give the desired concentration of inspired NO at a concentration of inspired oxygen ($FiO_2$) of 0.6–0.7. We used 60–70% oxygen to avoid hypoxic vasoconstriction.

*Procedure and data analysis:* The baseline ventilator settings were a fixed inflation volume ($\Delta V$) of 0.2 ± 0.01 (S.E.) l and a fixed inspiratory flow of 0.25 ± 0.01 (S.E.) l/s. Respiratory frequency was 24 ± 1 (S.E.) breaths/min. The ratio of inspiratory time to total breathing cycle duration was 0.33 ± 0.01 (S.E.) The baseline settings were kept constant throughout the experiment for all animals. To reduce the effects of the compliance of the system on the mechanics measurements, a fixed length standard low-compliance tube was used (2 cm i.d., 60 cm long) to connect the animal to the ventilator and the humidifier was omitted from the inspiratory line. The equipment flow resistance was 0.5 cm $H_2O$/l/s and the equipment dead space was 29.5 ml. Respiratory mechanics values were assessed by the constant $\mathring{V}$ end-inspiratory occlusion method.[20] Briefly, maintaining flow at its baseline value, single breath airway occlusions were performed randomly at inflation volumes between 0.05 and 0.2 l. Each occlusion was followed by a rapid initial drop in tracheal pressure ($P_{max} - P_1$) and was maintained until the apparent plateau ($P_2$) was achieved (5–6 s). The plateau represented the end-inspiratory elastic recoil pressure. The contribution of reduction in pressure during this period to continuing gas exchange is negligible. The initial tracheal pressure drop from $P_{max}$ to $P_1$, divided by the immediately preceding steady $\mathring{V}$, provided the interrupter resistance of the total respiratory system ($R_{int}$). Dividing $P_1 - P_2$ by $\mathring{V}$ before the occlusion, we obtained the additional effective resistance of the respiratory system ($\Delta R$) due to the viscoelastic properties of the thoracic tissues and the time constant inequalities within the system. The total resistance of the respiratory system ($R_{max}$) was obtained by dividing $P_{max} - P_2$ by the pre-occlusion flow. The static elastance of respiratory system ($E_{st}$) was obtained by dividing $P_2$ by the inspiratory volume, while the dynamic elastance ($E_{dyn}$) was obtained by dividing $P_1$ by pre-occlusion $V_t$.

*Statistical analysis:* Regression analysis was done by the least-squares method. The control values were compared with those obtained at the peak effect of ET-1 administration or after NO inhalation, using the Student two-tailed *t*-test. $p < 0.05$ was accepted as statistically significant. Values are means ± S.E.

### Results

Administration of ET-1 increased the mean pulmonary arterial pressure (from 18.88 ± 1.88 (S.E.) to 27.52 ± 3.49 (S.E.) mmHg) and the mean arterial pressure (from 113.06 ± 10.42 (S.E.) to 133.07 ± 10.87 (S.E.) mmHg), without a change in heart rate (HR). Inhalation of NO at the peak effect of ET-1 administration (about 10 min) significantly decreased MPAP (from 27.52 ± 3.43 (S.E.) to 16.95 ± 3.53 (S.E.) mmHg), without modifying systemic arterial pressure or heart rate. ET-1 administration and inhaled NO did not change haemoglobin, methaemoglobin or blood gas values (data not reported).

The *a* and *b* constants (intercept and slope) of the regression lines of $R_{max}$, $R_{int}$ and $\Delta R$ with $\Delta V$ in control condition, after ET-1 administration and after NO inhalation are given in Table 1. Figure 1 illustrates the mean relationships (± S.E.) between the respiratory resistances and $\Delta V$. ET-1 administration did not alter the positive correlation of $R_{max}$, $R_{int}$ and $\Delta R$ with $\Delta V$, but significantly increased the slope of the $\Delta R$ relationship. Inhalation of NO at the time of the peak effect of ET-1 administration (about 10 min) counterbalanced the peptide's effects on respiratory resistances, significantly changing the intercept and slope of $R_{max}$, $R_{int}$ and $\Delta R$ regression lines with $\Delta V$ (Table 1, Fig. 1).

Fig. 2 shows the mean relationships ± S.E. for $E_{st}$ or $E_{dyn}$ to $\Delta V$ in control conditions, after ET-1 administration and after NO inhalation. The data show that ET-1 did not alter the negative correlation of static

**Table 1.** Linear regressions obtained between the overall respiratory resistances ($R_{max}$), interrupter resistance ($R_{int}$) and additional resistance ($\Delta R$) and inflation volume ($\Delta V$, l).

| Pig (reference no.) | $R_{max}$ | | | | | | | | |
|---|---|---|---|---|---|---|---|---|---|
| | Ctr | | | ET-1 | | | NO | | |
| | *a* | *b* | *p* | *a* | *b* | *p* | *a* | *b* | *p* |
| 1 | 4.93 | 85.80 | 0.001 | 5.31 | 91.41 | 0.010 | 8.62 | 32.52 | NS |
| 2 | 6.10 | 54.25 | 0.001 | 4.41 | 82.71 | 0.001 | 5.87 | 67.12 | 0.001 |
| 3 | 1.32 | 90.40 | 0.050 | 1.65 | 91.65 | 0.001 | 2.13 | 78.06 | 0.001 |
| 4 | 2.00 | 62.19 | 0.001 | 2.41 | 94.51 | 0.001 | 6.46 | 67.61 | 0.001 |
| 5 | 5.39 | 71.30 | 0.001 | 7.15 | 68.95 | 0.001 | 9.29 | 54.50 | 0.001 |
| 6 | 14.55 | 39.29 | 0.050 | 12.84 | 51.97 | 0.001 | 14.22 | 34.41 | 0.005 |
| Mean | 5.72 | 67.21 | | 5.63 | 80.20 | | 7.77** | 55.70** | |
| S.E. | 2.33 | 27.44 | | 2.30 | 32.74 | | 3.17 | 22.74 | |

| Pig (reference no.) | $R_{int}$ | | | | | | | | |
|---|---|---|---|---|---|---|---|---|---|
| | Ctr | | | ET-1 | | | NO | | |
| | *a* | *b* | *p* | *a* | *b* | *p* | *a* | *b* | *p* |
| 1 | 3.69 | 34.92 | 0.001 | 5.24 | 29.76 | 0.001 | 6.41 | 8.10 | NS |
| 2 | 3.47 | 24.39 | 0.005 | 2.29 | 34.86 | 0.001 | 3.52 | 25.12 | 0.003 |
| 3 | 1.94 | 27.61 | 0.001 | 1.96 | 32.16 | 0.001 | 2.56 | 23.16 | 0.010 |
| 4 | 2.16 | 20.40 | 0.003 | 2.64 | 25.81 | 0.050 | 3.94 | 18.19 | 0.001 |
| 5 | 4.02 | 31.96 | 0.001 | 4.95 | 23.28 | 0.002 | 5.48 | 22.16 | 0.001 |
| 6 | 13.63 | 14.24 | 0.001 | 12.85 | 17.72 | 0.001 | 13.77 | 0.76 | NS |
| Mean | 4.82 | 25.59 | | 4.99 | 27.27 | | 5.95** | 16.25** | |
| S.E. | 1.97 | 10.45 | | 2.04 | 11.13 | | 2.43 | 6.63 | |

| Pig (reference no.) | $\Delta R$ | | | | | | | | |
|---|---|---|---|---|---|---|---|---|---|
| | Ctr | | | ET-1 | | | NO | | |
| | *a* | *b* | *p* | *a* | *b* | *p* | *a* | *b* | *p* |
| 1 | 1.24 | 50.88 | 0.001 | 0.06 | 61.65 | 0.050 | 2.23 | 24.41 | NS |
| 2 | 2.63 | 29.86 | 0.007 | 2.12 | 47.85 | 0.007 | 2.34 | 41.99 | 0.007 |
| 3 | -0.62 | 62.79 | 0.001 | -0.30 | 59.53 | 0.001 | -0.43 | 54.91 | 0.001 |
| 4 | -0.15 | 41.78 | 0.002 | -0.23 | 68.69 | 0.002 | 2.52 | 49.41 | 0.001 |
| 5 | 1.36 | 39.34 | 0.001 | 2.19 | 45.66 | 0.001 | 3.80 | 32.34 | 0.002 |
| 6 | 0.92 | 25.04 | 0.001 | -0.01 | 34.25 | 0.001 | 0.45 | 33.65 | 0.001 |
| Mean | 0.90 | 41.62 | | 0.64 | 52.94* | | 1.82** | 39.45** | |
| S.E. | 0.37 | 16.99 | | 0.26 | 21.61 | | 0.74 | 16.11 | |

Intercept (*a*, cmH'O.l$^{-1}$.s), slope (*b* cmH$_2$O.l$^{-1}$.s) and *p* of the curves. Ctr = control; ET-1 = endothelin-1 administration; NO = nitric oxide inhalation. The asterisks (*) indicate statistically significant differences of Ctr *vs.* ET-1 ($p < 0.05$); (**) indicate statistically significant differences of ET-1 *vs.* NO ($p < 0.05$).

and dynamic elastance with inflation volume but, even though not significantly, increased these values, especially when the respiratory system was inflated with smaller volumes. Inhalation of NO reduced the effects of ET-1 on static and dynamic elastance.

### Discussion

This study shows that inhaled nitric oxide can act as a selective local pulmonary vasodilator and as a bronchodilator. In the pig, inhaled NO reduces the pulmonary hypertension and the bronchoconstrictor effects of ET-1.

In spontaneously breathing lambs and in human volunteers with induced hypoxia, Frostell *et al.*[17,18] demonstrated that inhalation of NO at small doses (5–80 ppm) reversed hypoxic pulmonary vasoconstriction, without causing systemic vasodilatation, and attenuated thromboxane-mediated pulmonary vascular contraction. Consequently, it has been suggested that inhaled NO might be an important tool for reducing pulmonary hypertension in severe cardiac pulmonary diseases.[21] As in previous studies,[17,18] the present results show that in the pig, too, inhaled NO has a selective vasodilator activity in the lung, without affecting systemic pressure. This local response is probably due to its rapid reaction with haemoglobin to form methaemoglobin or to its conversion into nitrites and nitrates.[4,22,23] The rapid inactivation prevents the systemic vascular relaxing effect of NO, without altering the systemic vasoconstrictor activity of endothelin-1.

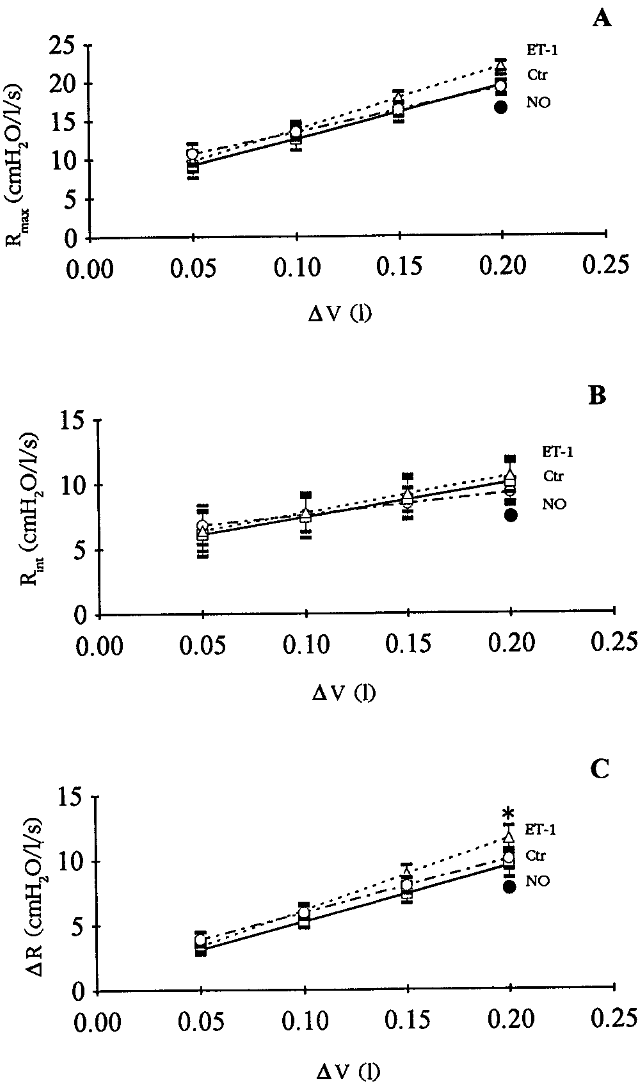

FIG. 1. Average ± S.E. ($n = 6$) relationships to ΔV of overall respiratory resistance ($R_{max}$) in Panel A; interrupter resistance ($R_{int}$) in Panel B; and additional resistance (ΔR) in Panel C of the respiratory system. Ctr, control, (——); ET-1 bolus administration, (----); NO inhalation, (—-—). The asterisks (*) indicate statistically significant differences of Ctr *vs.* ET-1 ($p < 0.05$); (•) indicate statistically significant differences of ET-1 *vs.* NO ($p < 0.05$).

The absence of significant changes in methaemoglobin, in our pigs, suggests that NO has modest toxicity when inhaled at low concentrations. As found by Frostell *et al.*[17] for spontaneously breathing lambs, inhalation of 80 ppm NO at $FiO_2$ of 0.6–0.7 caused a minimal oxidation of nitric oxide to $NO_2$. At high concentrations $NO_2$ causes pulmonary injury and bronchoconstriction.[24] We did not measure plasma levels of $NO_2$ and, although it is not possible to exclude the possibility that it might act on airway smooth muscles, our results show that inhalation of NO causes bronchodilatation, reducing the effects of ET-1 on respiratory resistances and elastance, relaxing precontracted airway smooth muscles.

The ratio of released ET-1 to NO regulates the vascular tone[25] and impairment of the ratio of production of these endothelium-derived factors could contribute to pulmonary hypertension associated with bronchoconstriction.

It has also been proposed that the mediators released by airway epithelium are important for regulation of bronchial tone. The epithelial lining of the airway produces an epithelium-derived relaxing factor (EpDRF),[26] considered to be similar to NO, and parenchyma lung is directly involved in release and clearance of ET-1.[11,12] The removal of epithelium induces hyperreactivity of the canine airway strip and changes the responsiveness of porcine bronchial smooth muscles.[27,28] It is also known that the mucus on the epithelial layer surface, binding oxygen radicals, acts as a limiting barrier for diffusion of NO. Even if this effect was present in our experiments, the observed reduction of ET-1-dependent vascular and respiratory effects shows that nitric oxide can diffuse throughout the epithelial layer. Even though in the pig ET-1 does not have a potent bronchoconstrictor activity, the inhaled NO relaxes smooth muscles and reduces respiratory resistances ($R_{max}$, $R_{int}$, ΔR). The endogenous release of nitric oxide due to peptide administration probably counteracts the true bronchoconstrictor activity of ET-1. Nitric oxide activity probably occurs only when the smooth muscles are precontracted because, as suggested by Dimori *et*

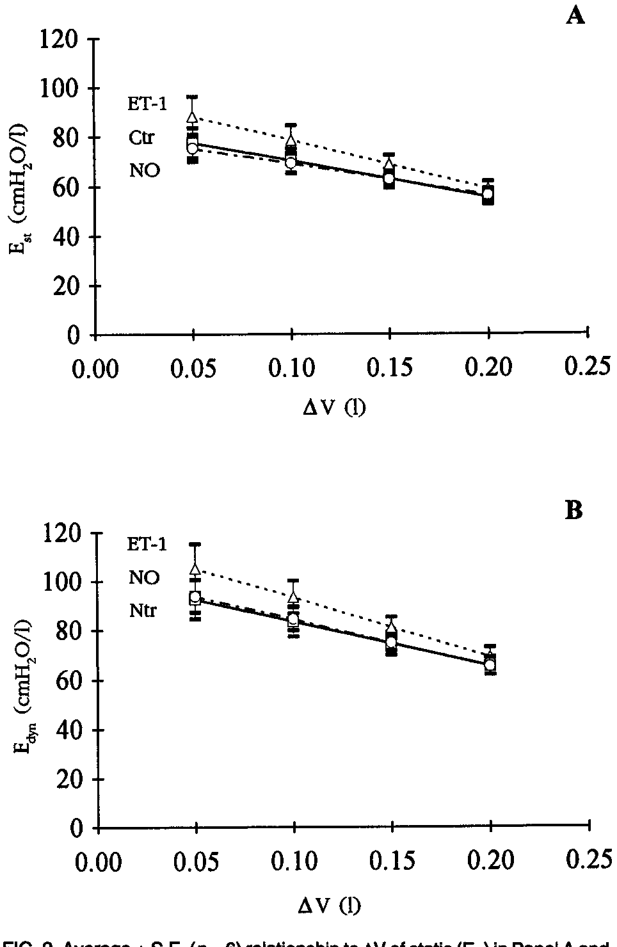

FIG. 2. Average ± S.E. ($n = 6$) relationship to ΔV of static ($E_{st}$) in Panel A and dynamic ($E_{dyn}$) in Panel B elastance of the respiratory system. Symbols are as in Fig. 1.

*al.*,[29] inhaled nitric oxide does not change normal bronchomotor tone.

It is known that there is an additional neural regulatory system of bronchial tone, besides the adrenergic and cholinergic system.[30,31] The mediators of this non-adrenergic non-cholinergic (NANC) system have not yet been identified although there is an increasing suspicion that NO is involved. Bronchial contraction due to methacholine[32] or electric field stimulation, which is known to act via the NANC system, is counterbalanced by NO.[33]

Recent studies have shown that when NO is added to tracheal and bronchial rings the tracheal rings relax whereas the bronchial rings are less dilated.[34] Its selective activity may have influenced the bronchodilating response observed in this study. Pison *et al.*[35] suggested that NO redistributes blood flow to the better-ventilated alveoli. This activity, together with reduction of respiratory resistances and elastance, may favour mechanical respiratory function and gas exchange. Also if our results show a modest activity of NO on ET-1 dependent bronchoconstriction they suggest therefore that inhaled nitric oxide may play an important therapeutic role in hypertensive and obstructive lung disease.

In summary, this study suggests that NO, when inhaled in small doses, does not have any significant toxicity, but has selective local pulmonary vasodilator and bronchodilator activity. Consequently, inhaled NO might well serve as an adjuvant therapy to existing treatment for bronchospastic and pulmonary hypertensive disorders, especially when they are caused by an imbalance of the ratio of endothelin-1 to nitric oxide.

### References

1. Yanagisawa M, Kurihara H, Kimura S, *et al.* A novel potent vasoconstrictor peptide produced by vascular endothelial cells. *Nature (Lond)* 1988; **332**: 411–415.
2. Uchida Y, Ninomiya H, Saotome M, *et al.* Endothelin a novel vasoconstrictor peptide, as a potent bronchoconstrictor. *Eur J Pharmacol* 1988; **154**: 227–228.
3. Furchgott RF, Zadawski JV. The obligatory role of endothelial cells in the relaxation of arterial smooth muscle by acetylcholine. *Nature* 1980; **288**: 373–376.
4. Palmer RMJ, Ferrige AG, Moncada S. Nitric oxide release accounts for the biological activity of endothelium-derived relaxing factor. *Nature* 1987; 327: 524–526.
5. Ignarro LJ, Buga GM, Wood KS, Byrns RE. Endothelium-derived relaxing factor produced and released from artery and vein is nitric oxide. *Proc Natl Acad Sci* 1987; **84**: 9265–9269.
6. Moncada S, Palmer RMJ, Higgs EA. Nitric oxide: physiology, pathophysiology and pharmacology. *Pharmacological Rev* 1991; **43**: 108–142.
7. Lerman A, Hildebrand FL, Margulies KB, *et al.* Endothelin: a new cardiovascular regulatory peptide. *Mayo Clin Proc* 1990; **65**: 1441–1454.
8. White SR, Hathaway DP, Umans JG, Leff AR. Direct effects on airway smooth muscle contractile response caused by endothelin-1 in guinea pig trachealis. *Am Rev Respir Dis* 1992; **145**: 491–493.
9. Battistini B, Filep J, Sirois P. Potent thromboxane-mediated bronchoconstrictor effect of endothelin in the guinea pig *in vitro*. *Eur J Pharmacol* 1990; **178**: 141.
10. Grunstein MM, Chuang ST, Schramm CM, Pawlowski NA. Role of endothelin-1 in regulating rabbit airway contractility. *Am J Physiol* 1991; **260**: L75–L82.
11. Pernow J, Hemsen A, Lundberg JM. Tissue specific distribution, clearance and vascular effects of endothelin in the pig. *Biochem Biophys Res Comm* 1989; **161**: 647–653.
12. Sirvio M-L, Metsarinne K, Saijonmaa O, Fyhrquist F. Tissue distribution and half-life of $^{125}$I-endothelin in the rat: importance of pulmonary clearance. *Biochem Biophys Res Comm* 1990; **167**: 1191–1195.
13. Belvisi MG, Stretton CD, Yacoub M, Barnes PJ. Nitric oxide is the endogenous neurotransmitter of bronchodilator nerves in humans. *Eur J Pharmacol* 1992; **210**: 221–222.
14. Munakata M, Masaki Y, Sahuna I, *et al.* Pharmacological differentiation of endothelium-derived relaxing factor from nitric oxide. *J Appl Physiol* 1990; **69**: 665–670.
15. Tucker JF, Brane SR, Charalambons L, Hoobbs AJ, Gibson A. L-N$^G$-Nitro arginine inhibits non-adrenergic, non-cholinergic relaxation of guinea pig isolated tracheal smooth muscle. *Br J Pharmacol* 1990; **100**: 663.
16. McLarty AJ, McGregor CGA, Miller VM. Endothelium-derived factors modulate contraction of bronchial smooth muscle. *Am J Physiol* 1993; **264**: R999–R1003.
17. Frostell CG, Fratacci M-D, Wain JC, Jones R, Zapol W. Inhaled nitric oxide, a selective pulmonary vasodilator reversing hypoxic pulmonary vasoconstriction. *Circulation* 1991; **83**: 2038–2047.
18. Frostell CG, Blomqvist H, Lundberg J, Hedenstierna G, Zapol WM. Inhaled nitric oxide dilates human hypoxic pulmonary vasoconstriction without causing systemic vasodilation. *Anesthesiology* 1991; **75**(suppl): A 989.
19. Pepke-Zaba J, Higenbottam TW, Dinh-Xuan AT, Stone D, Wallwork J. Inhaled nitric oxide as a cause of selective pulmonary vasodilation in pulmonary hypertension. *Lancet* 1991; **338**: 1173–1174.
20. Bates JHT, Rossi A, Milic-Emili J. Analysis of the behaviour of the respiratory system with constant inspiratory flow. *J Appl Physiol* 1985; **58**: 1840–1848.
21. Rossaint R, Falke KJ, Lopez F, Slama K, Pison U, Zapol WM. Inhaled nitric oxide for the adult respiratory distress syndrome. *N Engl J Med* 1993; **328**: 399–405.
22. Wennmalm A, Benthin G, Petersson AS. Dependence of the metabolism of nitric oxide (NO) in healthy whole blood on the oxygenation of its red cell haemoglobin. *Br J Pharmacol* 1992; **106**: 507–508.
23. Calver A, Collier J, Vallance P. Nitric oxide and cardiovascular control. *Experimental Physiol* 1993; **78**: 303–326.
24. Aguggini G, Albertini M. The reaction of $NO_2$ on respiratory system of the pig, evaluated by airway occlusion during constant flow and volume inflation. (Submitted).
25. Luscher TF, Yang Z, Tschudi M, *et al.* Interaction between endothelin-1 and endothelium-derived relaxing factor in human arteries and veins. *Circ Res* 1990; **66**: 1088–1094.
26. Munakata M, Masdaki Y, Ukita H, Homma Y, Kawakami Y. Is epithelium-derived relaxing factor (EpDRF) also nitric oxide (NO)? *Am Rev Resp Dis* 1989; **139**(suppl): A351.
27. Flavahan NA, Aarhus LL, Rimele TJ, Vanhoutte PM. Respiratory epithelium inhibits bronchial smooth muscle tone. *J Appl Physiol* 1985; **58**: 834–838.
28. Stuart-Smith K, Vanhoutte PM. Airway epithelium modulates the responsiveness of porcine bronchial smooth muscle. *J Appl Physiol* 1988; **65**: 721–727.
29. Dimori M, Clement MG. Effects of inhaled nitric oxide (NO) on the vascular and respiratory sysems of the pig. (In press).
30. Richardson JB. Nonadrenergic inhibitory innervation of the lung. *Lung* 1981; **159**: 315–322.
31. Lundberg JM, Saria A, Lundblad L, *et al.* Bioactive peptides in capsaicin-sensitive C-fiber afferents of the airways: functional and pathophysiological implications. In: Kaliner M, Barnes PJ, eds. *The Airways: Neural Control in Health and Disease.* New York: Marcel Decker, 417–445.
32. Dupuy PM, Shore SA, Drazen JM, Frostell CG, Hill WA, Zapol WM. Bronchodilator action of inhaled nitric oxide in guinea pigs. *J Clin Invest* 1992; **90**: 421–428.
33. Watson N, Maclagan J, Barnes PJ. Vagal control of guinea pig tracheal smooth muscle: lack of involvement of VIP or nitric oxide. *J Appl Physiol* 1993; **74**: 1964–1971.
34. Masaki Y, Munakata M, Ukita H, Homma Y, Kawakami Y. Nitric oxide (NO) can relax canine airway smooth muscles. *Am Rev Respir Dis* 1989; **139**(suppl): A 350.
35. Pison U, Lopez FA, Heidelmeyer CF, Rossaint R, Falke KJ. Inhaled nitric oxide reverses hypoxic pulmonary vasoconstriction without impairing gas exchange. *J Appl Physiol* 1993; **74**: 1287–1292.

**Received 1 November 1993; accepted in revised form 20 December 1993**
